# Comparing Postoperative Feeding Strategies in Elective Cholecystectomy Patients Under General Anesthesia: A Prospective Randomized Trial

**DOI:** 10.7759/cureus.73012

**Published:** 2024-11-04

**Authors:** Yangzin Lamo, Yashwant Singh Payal, Navin Kumar

**Affiliations:** 1 Anaesthesiology, Postgraduate Institute of Medical Education and Research (PGIMER), Chandigarh, IND; 2 Anaesthesiology, All India Institute of Medical Sciences Rishikesh, Dehradun, IND; 3 General Surgery, All India Institute of Medical Sciences Rishikesh, Dehradun, IND

**Keywords:** elective cholecystectomy, on-demand feeding, on-time feeding, patient’s satisfaction, postoperative care, postoperative feeding

## Abstract

Background and aims

Commencing early oral feeding soon after surgery is a crucial element of enhanced recovery after surgery (ERAS). However, it is imperative to ensure safety with a natural progression to oral intake and patient satisfaction. In this prospective, randomized, outcome assessor-blind controlled study, we aim to compare the efficacy of postoperative feeding on-demand versus predetermined timing in patients undergoing elective cholecystectomy under general anesthesia.

Materials and methods

A total of 120 patients (18 to 60 years, either gender) with American Society of Anaesthesiologists (ASA) physical status I-II, meeting inclusion criteria, and from the Department of General Surgery and Surgical Gastroenterology, were randomly allocated into two groups of 60 patients each. In the On-Demand group (Group OD), upon their request, patients received 200 ml of coconut water orally in small aliquots as per their comfort once fully awake. In the Predetermined-Time group (Group OT), patients received the same volume of coconut water at four hours post-surgery. The time of initial oral intake in Group OD, the incidence of postoperative nausea and vomiting (PONV), and patient satisfaction in both groups were assessed.

Results

There was no difference in the incidence of PONV between Group OD (13; 21.7%) and Group OT (15; 25%) (p=0.666). The majority of patients in both groups, 39 (65%) in Group OD and 40 (68%) in Group OT requested food within the initial ≤ 2 hours. Furthermore, overall satisfaction was significantly higher in Group OD compared to Group OT (p < 0.001).

Conclusions

Allowing postoperative oral feeding upon patient request enhances satisfaction without compromising safety as compared to adhering to predetermined schedules.

## Introduction

Following the introduction of general anesthesia in 1846, preoperative and postoperative fasting protocols were implemented to mitigate the risk of pulmonary aspiration of gastric contents. As a result, patients have traditionally abstained from oral intake after surgery until bowel function resumes. However, this practice has been linked to extended hospital stays and higher patient costs [1], which is not conducive to daycare surgery. The enhanced recovery after surgery (ERAS) protocol and the guidelines established by the European Society of Parenteral and Enteral Nutrition (ESPEN2017) have standardized this approach by reducing preoperative fasting periods and implementing early postoperative oral feeding. This has resulted in improvement in postoperative outcomes without a concurrent increase in postoperative readmission rates [2,3]. Reports have shown the safety and good tolerance of early oral feeding post-surgery, challenging conventional fasting practices until bowel function returns [4], Numerous studies support the feasibility and benefits of starting early oral intake after surgery [5-7].

Laparoscopic cholecystectomy is a common surgical procedure for symptomatic gallbladder disease. However, it frequently leads to postoperative nausea and vomiting (PONV), with incidence ranging from 30% to 80% [8,9]. With advancements in anesthesia practice, surgical techniques, and the ERAS protocol emphasizing early postoperative feeding, laparoscopic cholecystectomy has evolved into an outpatient procedure. Consequently, in most surgical procedures, the approach is shifting from traditional postoperative feeding to early oral intake. Some studies even suggest "ultra-early" postoperative feeding [10]. While early postoperative feeding is widely supported, the question of ‘how early?’ remains unanswered.

Previous studies have initiated oral feeding at predetermined postoperative times to assess its impact on PONV and postoperative satisfaction. We hypothesize that allowing patients to feed based on their desire aligns with a more natural and physiological approach, potentially reducing the incidence of nausea and vomiting and enhancing satisfaction rather than feeding at a predetermined time. Thus, we have designed this study to bridge the existing gap in the scientific literature concerning the effects of early postoperative feeding at conventional predetermined versus more physiological feeding based on the patient's demand.

## Materials and methods

This one-year, prospective, randomized, controlled, outcome assessor-blind study took place in a tertiary care institute having prior approval from the institutional ethical committee (IEC) of All India Institute of Medical Science (AIIMS) Rishikesh (Letter number AIIMS/IEC/21/38 dated 21-01-2021) and was registered in the Clinical Trial Registry of India (CTRI) vide no. CTRI/2021/03/031628. To determine the sample size for the primary objective, a previous study was considered, which assumed a clinically significant difference of 50% in the incidence of PONV after elective surgeries due to any intervention [11]. We utilized the data along with a 95% confidence level and 80% power to calculate the sample size, which came out to be 51 subjects in each group. Adjusting for a 20% non-response rate or loss to follow-up, we finally included 60 patients in each group. The study included individuals of both genders, aged 18-60 years, with American Society of Anesthesiologists (ASA) physical status grades I-II. Exclusion criteria comprised a body mass index (BMI) exceeding 30 kg/m^2^, a history of PONV, motion sickness, diabetes, hypertension, pregnancy, gastroesophageal reflux disease, renal disease, or any other systemic illness impacting gastric emptying. Additionally, patients undergoing chemotherapy, taking antiemetics, or using steroids were not considered for the study.

**Figure 1:**
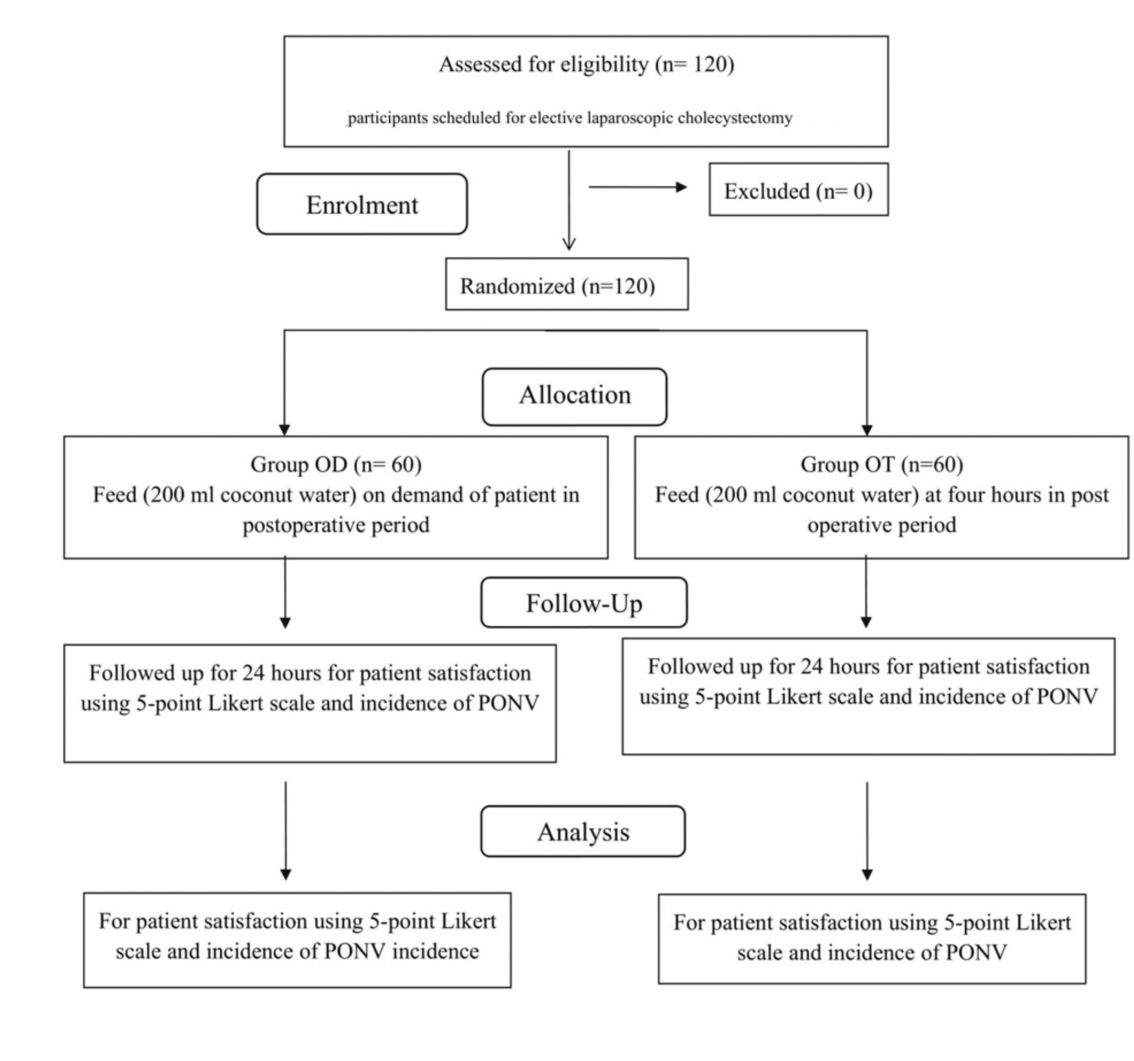
CONSORT flow diagram for randomized controlled trials CONSORT: Consolidated Standards of Reporting Trials; PONV: postoperative nausea and vomiting

Written informed consent was obtained from all eligible participants scheduled for elective laparoscopic cholecystectomy by the Departments of General Surgery and Surgical Gastroenterology. Using a sealed envelope technique, 120 patients were randomly assigned to either the study group “On-Demand” (Group OD) or the control group Predetermined Time, i.e. “On-Time” (Group OT) (Figure 1). During the postoperative phase, in the intervention group (Group OD), participants received 200 ml of coconut water orally upon the patient’s request, irrespective of the time. In contrast, Group OT patients received 200 ml of coconut water four hours after surgery as per institutional protocol. The primary outcome measure of the study was to assess the incidence of PONV and gauge patient satisfaction. Furthermore, the secondary outcome measure included determining the timing of the first oral intake in Group OD, as well as investigating the feasibility, clinical effectiveness, and safety of two different feeding schedules.

During preoperative visits, patients were instructed to abstain from oral intake for two hours for clear fluids and six hours for solid food before surgery. In the operating room, standard monitoring, including electrocardiogram (ECG), heart rate (HR), oxygen saturation (SpO2), and non-invasive blood pressure (NIBP), was applied to all patients. After securing an intravenous line, anesthesia induction involved the intravenous administration of fentanyl (1-2 μg/kg), propofol (1-2 mg/kg), and vecuronium (0.1 mg/kg). The airway was managed using a second-generation supraglottic airway device (SAD) of appropriate size. Neuromuscular monitoring (NMT) was maintained from anesthesia induction until the conclusion of surgery. Intravenous administration of a balanced salt solution was used to address the overall fluid deficit caused by fasting, manage any fluid losses during surgery, and provide ongoing maintenance fluid according to the patient's body weight.

The anesthesia was maintained with oxygen (40%) and air, with sevoflurane adjusted to achieve a minimum alveolar concentration (MAC) of 2. Intermittent boluses of vecuronium (0.05 mg/kg) were administered as needed. Parameters such as systolic blood pressure (SBP), diastolic blood pressure (DBP), mean arterial pressure (MAP), heart rate (HR), and oxygen saturation (SpO2) were documented before induction, every five minutes for the initial half-hour after induction of anesthesia, and subsequently every 10 minutes until the completion of the procedure. In the postoperative phase, patients' blood pressure, heart rate, and oxygen saturation were monitored hourly.

Hypotension was defined as a 25% reduction in MAP from the baseline, while bradycardia was characterized by an HR decrease below 50 beats per minute (bpm). Following surgery, neuromuscular blockade was reversed with intravenous neostigmine (0.03 mg/kg) and glycopyrrolate (0.01 mg/kg). Patients were then extubated and transferred to the post-anesthesia care unit (PACU). All patients were shifted to the PACU with maintenance intravenous fluid. 

Postoperatively, participants in Group OD were provided with oral intake, which included 200 ml of coconut water upon the patient’s request once they were fully awake and under the supervision of the nursing and anesthesia team, irrespective of the time. The time of the first oral feed was noted in Group OD. In Group OT, patients received the same volume of coconut water four hours after surgery. The coconut water was initially given in a small aliquot according to the patient’s comfort, followed by the remaining amount in both groups.

During the postoperative phase the time of first oral intake by the patient in Group OD, the total volume consumed by patients within the initial eight hours in both the groups, side effects, such as nausea and vomiting using the PONV impact scale Score [12], and patient's overall satisfaction using a 5-point Likert scale [13] with modifications (5-very much satisfied, 4-satisfied, 3-neither satisfied nor dissatisfied, 2-dissatisfied, and 1-very much dissatisfied) in both groups were evaluated at 24 hours after the surgery by an assessor who was otherwise not aware of the postoperative feeding status of the patient after receiving a call from the staff nurse in the PACU.

Statistical analysis

All the data were entered into an Excel spreadsheet (Microsoft Corporation, Redmond, WA, US) and were analyzed using the IBM SPSS version 24.0. software (IBM Corp., Armonk, NY, US). Continuous variables, such as age, weight, body mass index (BMI), heart rate, blood pressure, SpO2, and arterial blood gas (ABG) values were expressed as mean ± standard deviation (mean ±SD) with a 95% confidence interval (95 CI). These continuous variables were evaluated for normal distribution using the Kolmogorov- Smirnov test (KS) and were analyzed using the student’s t-test. Categorical data were presented as frequencies and percentages and analyzed using the chi-square or Wilcoxon-Mann-Whitney U test as appropriate. Considering a predetermined dropout rate, the intention-to-treat principle was employed in all analyses. A two-sided P value of <0.05 was considered indicative of statistical significance.

## Results

One hundred and twenty patients underwent eligibility assessments and were randomly allocated into Group OD (n = 60) and Group OT (n = 60) (Figure 1). The demographic characteristics, including age, weight, height, BMI, gender, ASA physical status, and duration of anesthesia and surgery, were comparable between the two groups (Table 1).

**Table 1:** Characteristics of the two groups of patients BMI: body mass index; ASA: American Society of Anaesthesiologists

	Group OD (n=60)	Group OT (n=60)	P value
Age in Years (mean ±SD)	43.52 ± 10.37	41.82 ± 11.95	0.407
Height in Centimeter (mean ±SD)	159.02 ± 6.70	158.67 ± 6.80	0.485
Weight in Kg (mean ±SD)	59.67 ± 6.48	58.96 ± 7.02	0.687
BMI in kg/m2 (mean ±SD)	23.61 ± 2.33	23.44 ± 2.67	0.564
Anaesthesia Time in Minutes (mean ±SD)	122.92 ± 30.48	122.25 ± 27.47	0.43
Surgical Time in Minutes (mean ±SD)	85.72 ± 19.26	82.00 ± 16.98	0.15
ASA I Number (%)	36 (60.0%)	43 (71.7%)	0.178
ASA II Number (%)	24 (40.0%)	17 (28.3%)	0.178

Regarding the occurrence of PONV, it was noted that 13 (21.7%) participants in Group OD and 15 (25.0%) in Group OT experienced it. However, this disparity did not reach statistical significance (p=0.666) (Table 2).

**Table 2:** Incidence of PONV between the two groups PONV: postoperative nausea and vomiting

PONV Episode	Group OD (n= 60)	Group OT (n= 60)	P value
Yes	13 (21.7%)	15 (25.0%)	0.666
No	47 (78.3%)	45 (75.0%)	

Patient satisfaction was evaluated using the Likert scale at 24 hours postoperatively. There was a significant difference between the two groups, with the median Likert scale score being the highest in Group OD. The median interquartile range (IQR) of the Likert scale score was significantly higher in Group OD 4 (4-5) as compared to Group OT 2 (2-3). The Likert scale score ranged from 3 to 5 in Group OD and from 1 to 3 in Group OT (W = 3871.000, p < 0.001), signifying a statistically significant difference. Furthermore, an analysis of the relationship between 'Group' and 'Likert scale' revealed a significant difference in Likert scale distribution among the groups. Notably, Group OD demonstrated a superior satisfaction level compared to Group OT (χ2 = 88.000, p < 0.001) (Table 3).

**Table 3:** Likert scale score at 24 hours between the two groups † Wilcoxon-Mann-Whitney U test (W); # chi-squared test (χ2); * Highly significant

Likert Scale Score	Group		Wilcoxon-Mann-Whitney U Test (W)/Chi-Squared Test (χ2)	
	Group OD (n=60)	Group OT (n=60)	3578.000^†^	P value* <0.001
Mean (SD)	4.01 (0.53)	2.60 (0.44)		
Median (IQR)	4 (4-5)	2 (2-3)		
Min - Max	03-May	01-Mar		
Score 1 n (%)	0 (0.0%)	0 (0.0%)	88.000 ^#^	P value* <0.001
Score 2 n (%)	0 (0.0%)	31 (51.66%)		
Score 3 n (%)	13 (21.66%)	29 (48.33%)		
Score 4 n (%)	20 (33.33%)	0 (0.0%)		
Score 5 n (%)	27 (45.00%)	0 (0.0%)		
Total n (%)	60 (100.0%)	60 (100.0%)		

**Figure 2:**
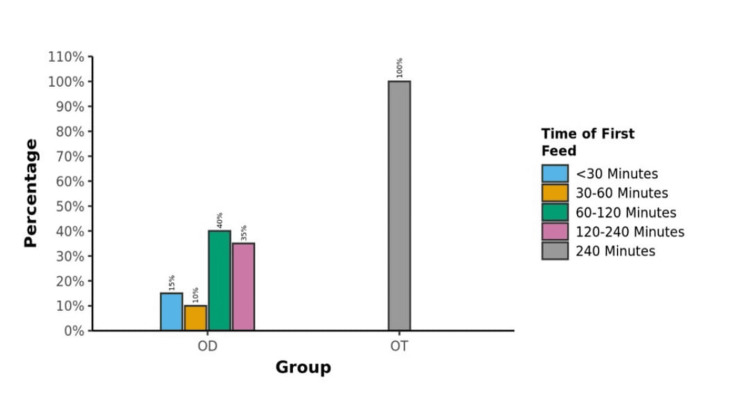
Time of first feed OD: Group OD; OT: Group OT

The timing of the first feeding was significantly earlier in Group OD as compared to Group OT (Figure 2). Specifically, 15% of patients asked for oral fluid in less than 30 minutes, 10% requested between 30 and 60 minutes, 40% requested between 60 and 120 minutes, and 35% of patients requested between 120 and 240 minutes. The majority of patients in both groups requested oral fluids within the first two hours, comprising 39 (65%) in Group OD and 40 (68%) in Group OT. However, because of protocol, the patients in Group OT were restricted from oral fluid intake until four hours postoperatively. Nevertheless, all patients in both groups received fluids by or at four hours postoperatively. There was no difference in the total volume consumed orally in the first eight hours between both groups postoperatively (p=0.417) (Table 4).

**Table 4:** Time of first feed (minutes) and total volume in the first eight hours † P value 0.476

Time of first feed	Group OD (n=60)	Group OT (n=60)
Less than 30 min	9 (15 %)	0%
30 to 60 min	6 (10 %)	0%
60 to 120 min	24 (40%)	0%
120 to < 240 min	21 (35%)	0%
≧240 min	0 (0%)	60 (100%)
Total volume consumed in first 8 hours in milliliter (mean± SD) †	390 ±35	386 ± 25

## Discussion

The current study highlights the feasibility of commencing early oral feeding based on the preferences of patients undergoing elective laparoscopic cholecystectomy. In this study, we used the term feeding “On-Demand" to signify that the feed is administered at the patient's request, regardless of predetermined postoperative timing based on the request of other than the patient in the PACU. Patients consumed 200 mL of coconut water in the PACU once fully awake, under the supervision of the nursing and anesthesia team. In cases where “On-Demand” feeding was not provided, the standard institutional postoperative protocol was followed, requiring a minimum of four hours of nil per oral (NPO) for liquids during the postoperative period.

In modern medicine, with advancements in anesthesia and a growing preference for performing surgical procedures on a daycare basis, the early initiation of oral feeding holds considerable significance. Early initiation of oral feeding in the postoperative period is one of the key aspects of ERAS and the protocol outlined by the ESPEN 2017 [2,3]. However, these protocols do not specify a fixed timeframe for commencing postoperative feeding. ACERTO guidelines similarly advocate for early oral or enteral feeding, provided the patient remains hemodynamically stable, even in cases like gastrointestinal anastomosis [14]. This recommendation extends to patients undergoing laparoscopic cholecystectomy, hernioplasty, and anorectal procedures, emphasizing the initiation of early oral diets and hydration without the necessity for intravenous hydration.

In the present study, we began oral feeding in the postoperative area at the patients’ request, adopting a more physiological approach to feeding on demand. Franco AC et al. also introduced early oral feeding in the postanesthetic recovery area (PAR), terming it "ultra-early" [10]. However, unlike their study, the decision to initiate early feeding in our study was made by the patient rather than the physician, which appears more logical. Though Franco AC et al. refer to this feeding as "ultra-early," the timing of the first feed was only specified as ‘oral feeding during post-anesthesia recovery’ (PAR). In our study, the majority of the patients (65%) commenced oral feeding within the initial two hours of the postoperative period. It's also important to note here that most of the patients (68%) in Group OT also expressed their willingness for oral fluids within the initial two hours postoperatively. However, due to methodological criteria, they were only permitted oral intake at a predetermined time (4 hours) postoperatively.

Previous studies have typically followed predetermined schedules for postoperative feeding, often initiated through physician orders [2,3,10,14]. In contrast, we propose an "On-Demand" approach, allowing patients to start oral feeding based on their own request and readiness. This approach looks more natural and has a physiological way of aligning with patient comfort levels and satisfaction. This patient-driven approach to postoperative feeding holds particular relevance for daycare procedures. Also, describing the postoperative feeding in terms of standard time gives a more meaningful message that can be given as a reference.

In our study, we found that the occurrence of PONV following laparoscopic cholecystectomy in both Group OD (21.7%) and Group OT (25%) was notably lower than the widely reported range of 30% to 80% [8,9]. Despite observing a trend toward lower PONV rates among patients who received oral feed upon request, this difference lacked clinical significance as compared to predetermined scheduled feeding times. The lower incidence of PONV in the present study may be attributed to the fact that we administered Propofol for induction, known for its antiemetic properties along with avoidance of emetogenic nitrous oxide.

Patient satisfaction plays a crucial role in assessing the holistic success of any surgical intervention. Our observations indicate that allowing patients to initiate oral feeding on their request enhances satisfaction without compromising safety compared to adhering to a fixed feeding schedule. Udayasankar M et al., in their study, observed the improvement in anxiety with overall perioperative comfort of the patient after the adoption of the ERAS approach [15]. Therefore, early feeding in the postoperative period holds considerable promise as a clinical strategy to enhance recovery among surgical patients. Allowing patients to feed at their request postoperatively also leads to higher satisfaction.

Limitations

The present study has some limitations. First, our research focused exclusively on laparoscopic cholecystectomy, which may have restricted the generalizability of our findings. Including a wider range of laparoscopic surgeries could have provided a more comprehensive replication of the study and potentially yielded improved outcomes. Second, we did not quantify the total intravenous fluid administered within the initial eight hours following surgery.

## Conclusions

This study demonstrates that on-demand oral feeding after elective laparoscopic cholecystectomy is both feasible and beneficial. Unlike the traditional approach of delaying oral intake, allowing patients to drink fluids when they feel ready did not increase the incidence of postoperative nausea and vomiting (PONV) but led to significantly greater patient satisfaction. Early oral intake also aligns with ERAS protocols, supporting faster recovery without raising the risk of adverse events. Our findings suggest that oral intake timing should be adjusted based on the patient's readiness rather than adhering strictly to a protocol-driven timeframe, supporting a more individualized, patient-centered approach in postoperative care. However, this reference time to commence oral feed may vary for other surgical procedures; hence, further research with larger sample sizes and diverse surgical populations is warranted to validate these findings, and current fasting guidelines can be revised accordingly.
